# The Impact of Inspiratory Muscle Training on Diaphragm Thickness in Healthy Adults: A Systematic Review and Meta-Regression

**DOI:** 10.3390/medicina62030609

**Published:** 2026-03-23

**Authors:** Cemre Didem Eyipınar, Tolga Altuğ, Mesut Süleymanoğulları, Aslıhan Tekin, Nicola Luigi Bragazzi, Valentina Stefanica, Halil İbrahim Ceylan

**Affiliations:** 1 Division of Physical Education and Sport, Department of Physical Education and Sport, Faculty of Sport Sciences, Gaziantep University, 27310 Gaziantep, Türkiye; cemreeyipinar@gantep.edu.tr; 2 Division of Coaching Education, Department of Coaching Education, Faculty of Sport Sciences, Ağrı İbrahim Çeçen University, 04100 Ağrı, Türkiye; taltug@agri.edu.tr; 3 Division of Physical Education and Sport, Department of Physical Education and Sport, Faculty of Sport Sciences, Ağrı İbrahim Çeçen University, 04100 Ağrı, Türkiye; msuleymanogullari@agri.edu.tr (M.S.); astekin@agri.edu.tr (A.T.); 4 Laboratory for Industrial and Applied Mathematics (LIAM), Department of Mathematics and Statistics, York University, Toronto, ON M3J 1P3, Canada; robertobragazzi@gmail.com; 5 Department of Physical Education and Sport, Faculty of Sciences, Physical Education and Informatics, National University of Science and Technology Politehnica Bucharest, Pitesti University Center, 060042 Pitesti, Romania; 6 Department of Physical Education of Sports Teaching, Faculty of Sports Sciences, Atatürk University, 25240 Erzurum, Türkiye

**Keywords:** respiratory muscle adaptation, inspiratory training modality, hypertrophy, diaphragm muscle morphology, ultrasonography

## Abstract

*Background and Objectives*: The hypertrophic adaptation of the diaphragm to inspiratory muscle training (IMT) remains insufficiently characterized, particularly in healthy and athletic populations. To address this gap, we conducted a meta-analysis and meta-regression to evaluate the effects of IMT on diaphragm thickness and identify potential moderating factors. *Materials and Methods*: A systematic search was conducted across PubMed, MEDLINE, Embase, CINAHL, and SPORTDiscus as well as Google Scholar (gray literature) through November 2025. Eight studies involving 203 healthy participants met the inclusion criteria. A random-effects model was used to calculate pooled effect sizes and meta-regression estimates. *Results*: IMT produced a statistically significant moderate increase in diaphragm muscle thickness, with a standardized mean difference (SMD) of Hedges’ g = 0.52 (95% CI: 0.19 to 0.85; *p* < 0.05). Subgroup analyses indicated that IMT with 50% maximal inspiratory pressure (MIP) produces a statistically significant effect (*p* = 0.0069), whereas fitness status and age did not significantly influence outcomes (*p* = 0.589 and *p* = 0.126, respectively). Meta-regression analyses revealed that only baseline MIP value (β = 0.030; 95% CI: 0.009 to 0.050; *p* = 0.004) was associated with diaphragm hypertrophy. *Conclusions*: IMT with 50% of MIP elicits meaningful diaphragmatic hypertrophy in healthy individuals. This response appears independent of fitness status or age, but is significantly influenced by baseline inspiratory muscle strength (MIP). These findings support the utility of IMT in enhancing respiratory muscle morphology in health and performance contexts.

## 1. Introduction

The diaphragm is a dome-shaped, musculotendinous structure that separates the thoracic and abdominal cavities and plays a central role in respiration. It consists of approximately equal proportions of slow- and fast-twitch muscle fibers, allowing it to dynamically adjust its thickness in response to variations in breathing depth [1,2].

Increased diaphragm thickness may improve athletic performance by increasing respiratory muscle strength and ventilatory efficiency. Inspiratory muscle training (IMT), a form of resistance exercise [3], has been proposed to enhance diaphragmatic hypertrophy, potentially delaying respiratory muscle fatigue and improving aerobic capacity, particularly during activities with high ventilatory demand, such as endurance sports [4].

Furthermore, investigating the effects of IMT on diaphragm muscle thickness is of paramount clinical importance for preventing diaphragmatic atrophy, frequently observed in chronic respiratory diseases (e.g., chronic obstructive pulmonary disease or COPD) and critically ill patients undergoing prolonged mechanical ventilation [5,6,7,8], and to monitor the pulmonary rehabilitation status of individuals with weakened diaphragm muscle due to stroke [9], neurological conditions [10], or heart failure [11]. In this context, investigating the potential clinical effects of IMT on diaphragm thickness could provide an objective, quantitative biomarker of treatment efficacy by demonstrating not only functional improvements in diaphragm capacity but also reversibility at the structural level [12].

Despite the growing body of scholarly literature on the function of the respiratory muscles, no systematic review or meta-analysis has specifically addressed the effects of IMT on diaphragmatic hypertrophy in healthy individuals and athletes. While IMT has been widely recognized as a beneficial intervention in clinical populations with respiratory conditions [13,14,15,16,17], its role in enhancing diaphragm thickness among non-clinical groups remains insufficiently understood.

The impact of IMT on diaphragm thickness has been the focus of three published meta-analyses to date [9,15,17]. However, only stroke patients were included in these meta-analyses. With a moderate degree of certainty, Liu et al. [9] found that IMT improved diaphragmatic function in stroke survivors (9 studies, N = 255). Based on the meta-analysis, IMT significantly increased diaphragm thickening fraction on both the affected (51%) and non-affected (37%) sides. Nevertheless, only two studies evaluating diaphragm thickness were included. Examining its impact on diaphragm muscle thickness was not feasible due to the small sample size. In a meta-analysis examining the effects of IMT after stroke (9 studies; N = 463), Fabero-Garrido et al. [17] reported that IMT resulted in a significant increase in diaphragm thickness (3 studies, standardized mean difference or SMD = 0.9, *p* < 0.05) in the short term. However, in this meta-analysis, the overall impact of IMT and expiratory muscle training (EMT) was the primary focus, and the individual impact of IMT was reported only as a subgroup analysis. In another meta-analysis examining the effects of IMT after stroke (13 studies; N = 373), Zhang et al. [15] reported that IMT significantly increased diaphragm thickness (4 studies, SMD = 0.47, *p* = 0.005). However, the quality of the included studies was not reported, and the inclusion criteria were unknown. Additionally, the diaphragmatic thickness ratio was calculated using different formulas, thereby precluding inter-study data integration.

The present meta-analysis assesses hypertrophic responses of IMT in the diaphragm in healthy and athletic populations, which have not been previously explored in the literature, using structured moderator analyses and multivariate meta-regression. As already mentioned, all previous reports focus solely on stroke patient groups: Liu et al. [9] included only a few studies; Fabero-Garrido et al. [17] did not examine the effect of IMT alone; and Zhang et al. [15] failed to report study quality.

As such, the literature cannot yet provide a single comprehensive synthesis focusing solely on the effect of IMT on the diaphragm, with high-quality studies examining healthy adults and athletes and systematically assessing numerous exercise-related moderators, such as exercise intensity and frequency, age, fitness status, and baseline lung capacities.

Therefore, to address this gap in the literature, the key objective of this study was to evaluate, through meta-analysis and meta-regressions, the effects of IMT on diaphragm thickness in healthy adults and to identify relevant moderating factors. We hypothesized that IMT elicits measurable increases in diaphragm thickness and that the magnitude of these adaptations varies with IMT. Given the limited number of direct assessments and methodological constraints in the existing literature, our findings may help optimize IMT protocols, particularly in athletic settings where enhanced ventilatory efficiency can improve performance.

## 2. Materials and Methods

### 2.1. Registration

The research protocol was registered on the Open Science Framework (OSF) prior to conducting the literature search (https://doi.org/10.17605/OSF.IO/9ZQMU). The reporting of these research findings followed the Preferred Reporting Items for Systematic Reviews and Meta-Analyses (PRISMA) criteria [18].

### 2.2. Eligibility Criteria

Studies were included if they met the following conditions:

- Investigated the effects of IMT on diaphragm muscle thickness.
- Included only healthy participants (no history of respiratory, cardiovascular, neurological, muscular, or metabolic problems; no drugs).
- Reported pre-post changes in site-specific hypertrophy using a validated imaging technique, such as B-mode ultrasonography.
- Designed as randomized controlled trials (RCTs) or pre-post studies, published as peer-reviewed articles.

Studies were excluded if they met any of the following conditions:

- Only studies reporting muscle thickness data for muscles other than the diaphragm.
- Included individuals with chronic diseases or animal models (e.g., rat studies).

### 2.3. Search Strategy

A comprehensive automated search was conducted across Google Scholar (gray literature), PubMed, Embase, MEDLINE, CINAHL, and SportDiscus, without language restrictions, covering publications from inception through November 2025. Boolean search operators were applied using the following keyword combinations: (“respiratory muscle training” OR “inspiratory muscle training” OR “inspiratory training” OR “respiratory training”) AND (diaphragm OR “diaphragm muscle thickness” OR “diaphragm hypertrophy”).

### 2.4. Data Extraction

The data from the included studies were systematically extracted and recorded in a Microsoft Excel spreadsheet, adhering to the following criteria:

- Research Characteristics—Study author and year of publication.
- Participant Demographics—Population details, including sample size, sex/gender, and health status.
- Training Methods—Intensity, sets, duration, and repetitions of IMT.
- Outcome Measures—Means and standard deviations of pre- and post-test measurements, focusing on diaphragm muscle thickness. The analysis included data on relaxed diaphragm thickness (i.e., thickness at end-expiration).

To address missing data on pre-post changes, the corresponding authors were contacted via email. At the initial stage, the relevant literature was systematically screened across multiple databases following a structured literature search strategy. The titles, abstracts, and keywords of the identified studies were independently reviewed by two investigators (CDE and TA) to ensure adherence to the predefined inclusion criteria.

Following this screening, qualitative and quantitative evaluations were conducted on the eligible studies. The final dataset included studies that met the specified criteria, covering the following four key aspects:

- Study Identification—Name of the study, year of publication, and participant demographics (age, sex/gender).
- IMT Protocol—Training procedures, duration, study population, and sample size.
- Outcome Measures—Changes in diaphragm muscle thickness.
- Measurement Methodology—The use of ultrasound for assessing diaphragm muscle thickness.

To ensure data integrity, the extracted findings were cross-checked twice among coders, and any discrepancies were resolved through mutual agreement.

### 2.5. Methodological Quality Assessment

In this systematic review and meta-analysis, the Physiotherapy Evidence Database (PEDro) scale was employed to assess the methodological quality of the included studies. The PEDro scale was developed by integrating the Delphi List [19], a nine-item expert-generated checklist, with two additional items related to statistical reporting.

The scale comprises eleven criteria, categorized as follows: external validity (Item 1), internal validity (Items 2–9), and statistical reporting (Items 10–11). Each item is scored as either “yes” (1 point) or “no” (0 points) based on whether the study explicitly met the specified criterion. Higher scores indicate superior methodological quality. Studies scoring 4 points are classified as “poor”, those scoring 4 to 5 points as “fair”, scores between 6 and 8 points are considered “good”, and studies scoring 9 to 10 points are categorized as “excellent” in methodological quality [20].

### 2.6. Statistical Analysis

The R software (v. 4.4.2; R Foundation for Statistical Computing, Vienna, Austria) metafor package was used to conduct a random-effects meta-analysis to evaluate the effect of IMT on diaphragm thickness. SMDs and 95% confidence intervals (CIs) were calculated from the obtained pre- and post-diaphragm thickness (mm) means and standard deviations [21]. Missing post-intervention SDs were imputed using a correlation coefficient of 0.7 between pre- and post-measurements, as recommended for post-score calculations by the Cochrane Handbook for Systematic Reviews of Interventions [22]. Heterogeneity was evaluated using the I^2^ statistic. I^2^ > 50% was considered indicative of high heterogeneity. To assess the relationships among IMT intensity, age, baseline pulmonary volumes (forced expiratory volume in 1 s [FEV1], forced vital capacity [FVC], maximal inspiratory pressure [MIP], and maximal expiratory pressure [MEP]), and diaphragm muscle thickness, meta-regression analyses were conducted in R. The PEDro scale was used to assess study quality, and the funnel plot was used to detect publication bias; however, these analyses should be interpreted with caution given the small number of included studies. The significance threshold was set at *p* ≤ 0.05 for all outcomes.

## 3. Results

### 3.1. Literature Search and Study Selection

**Figure 1:**
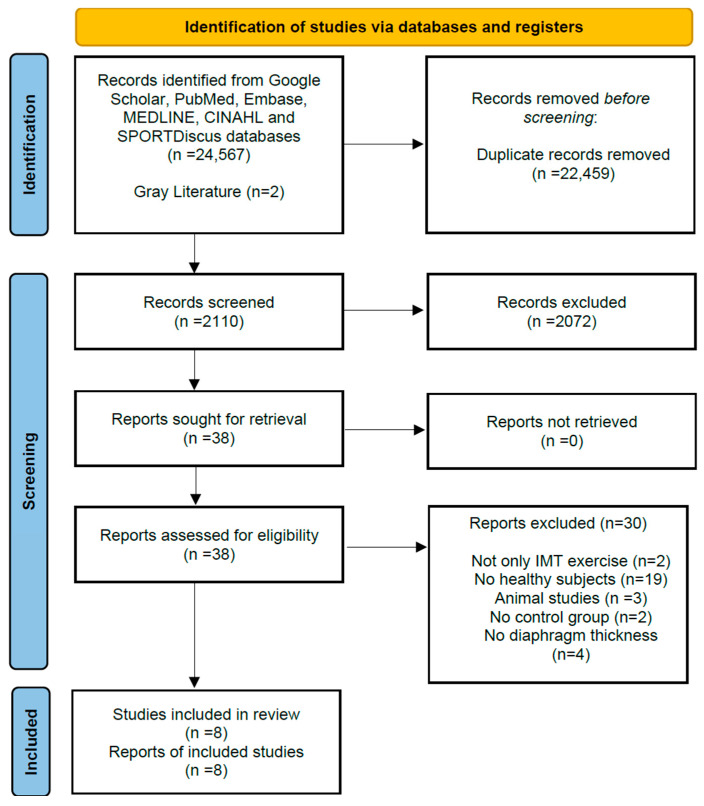
Preferred Reporting Items for Systematic Reviews and Meta-Analyses (PRISMA) Flow Chart of Study Selection Process [18] (see Supplementary Materials).

The initial search retrieved 24,569 articles. After removing duplicate records, 2110 articles remained. Of these, 2072 studies were excluded based on the following criteria: studies focusing on different muscle groups (e.g., accessory inspiratory muscles), studies incorporating additional interventions, and studies assessing muscle activation via electromyography (EMG) rather than ultrasonography. Following these exclusions, 38 studies met the preliminary eligibility criteria for synthesis. Subsequently, an additional 30 studies were excluded for reasons detailed in Figure 1. Ultimately, a total of 8 studies were included in this systematic review and meta-analysis.

### 3.2. Study Characteristics

The present systematic review and meta-analysis comprised 203 healthy individuals (97 female and 106 male) from 8 trials. Each study had a different sample size, ranging from 10 to 80 individuals. Across trials, respiratory muscle training intensities ranged from 30% to 80% of MIP. The training duration ranged from 4 to 8 weeks; in general, it was 8 weeks. 

### 3.3. Quality Assessment

The findings of the research quality assessment, including the PEDro Scale results, are presented in Table 1.

The mean publication-quality score for the studies was 7.37, indicating an overall good level. Based on the PEDro scale, all of the studies were of high quality (PEDro score ≥ 6).

### 3.4. Categorization of Studies

Table 2 lists the studies included in the present systematic literature review.

### 3.5. Heterogeneity

The analysis is based on eight studies. Heterogeneity among the individual studies identified in this study was assessed using Cochran’s Q test and the I^2^ statistic to evaluate inconsistencies among study results [31]. The heterogeneity test table used to determine the preferred method is presented below (Table 3).

The random-effects meta-analysis of eight studies demonstrated a significant overall effect (Hedges’ g = 0.52, SE = 0.17, 95% CI [0.19, 0.85], z = 3.07, *p* = 0.002). The estimated heterogeneity was moderate (τ^2^ = 0.095, I^2^ = 45.2%), and the heterogeneity test was not statistically significant (Q(7) = 12.71, *p* = 0.080), suggesting that the variability among studies was moderate and that the pooled effect size was consistent across the included studies.

### 3.6. Publication Bias

**Figure 2:**
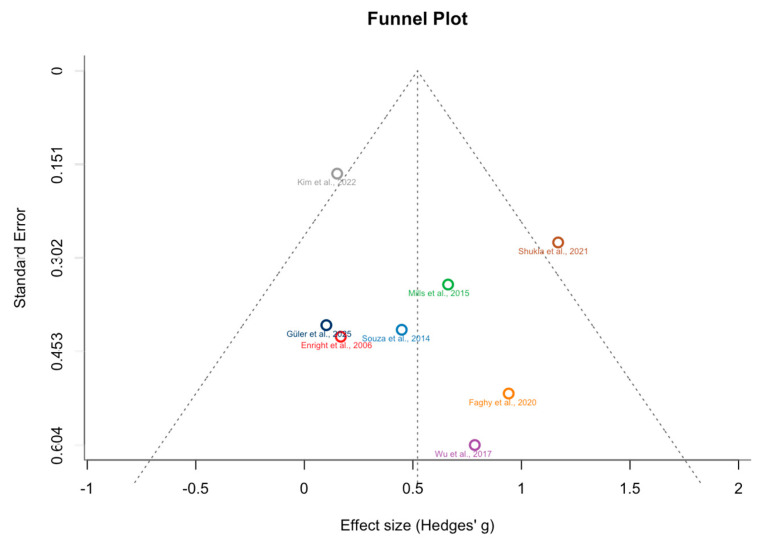
Funnel Plot (publication bias analysis of the studies) [23,24,25,26,27,28,29,30].

When the funnel plot in Figure 2 is analyzed, a symmetrical distribution of effect sizes around the mean indicates no evidence of publication bias. This conclusion was supported by formal tests: the Rank Correlation Test (Begg’s test) yielded Kendall’s tau = 0.143 (*p* = 0.720), and Egger’s regression test was also nonsignificant (z = 0.494, *p* = 0.621), indicating no small-study effects. Additionally, the Trim-and-Fill procedure estimated zero missing studies on the left side, confirming the robustness of the overall effect size (Hedges’ g = 0.521, 95% CI [0.188, 0.855], *p* = 0.0022). The random-effects model indicated moderate heterogeneity (tau^2^ = 0.095, I^2^ = 45.2%, Q(7) = 12.71, *p* = 0.080), and the funnel plot further supported the symmetry of the data, suggesting that the observed significant effect is unlikely to be influenced by publication bias.

**Figure 3:**
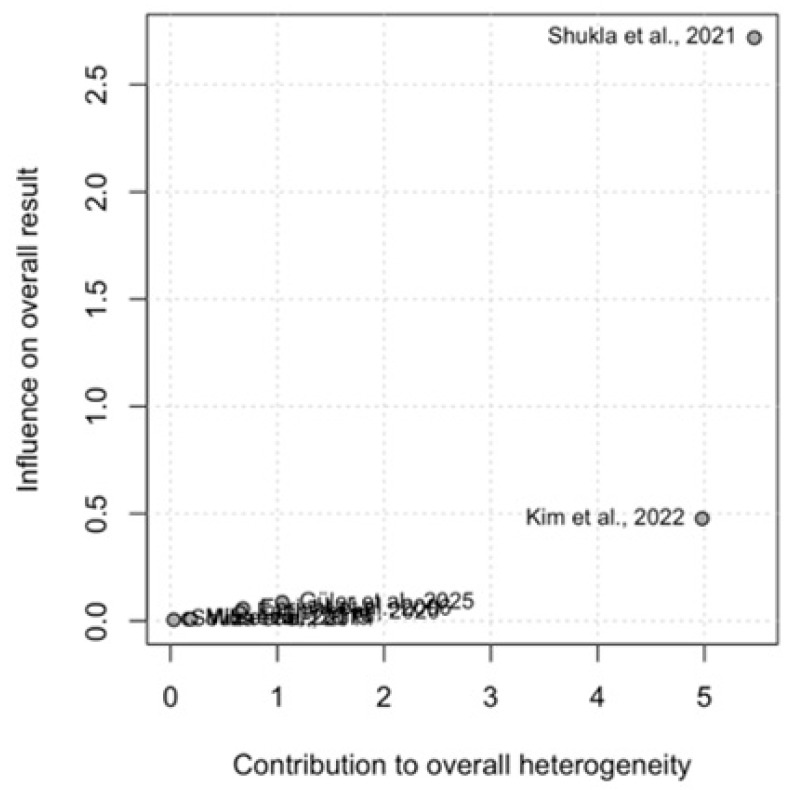
Baujat Plot (studies’ contributions to overall heterogeneity) [23,24,25,26,27,28,29,30].

The Baujat results in Figure 3 indicate that Shukla et al., 2021 [28] and Kim et al., 2022 [29] have the largest influence on both heterogeneity and effect size, while Enright et al., 2006 [23], Faghy et al., 2021 [27], and Güler et al., 2025 [30] show moderate contributions. Souza et al., 2014 [24], Mills et al., 2015 [25], and Wu et al., 2017 [26] contribute minimally, suggesting that the overall meta-analysis heterogeneity is largely driven by the Shukla et al., 2021 [28] and Kim et al., 2022 [29] studies.

### 3.7. Effect of Inspiratory Muscle Training on Diaphragm Thickness

**Figure 4:**
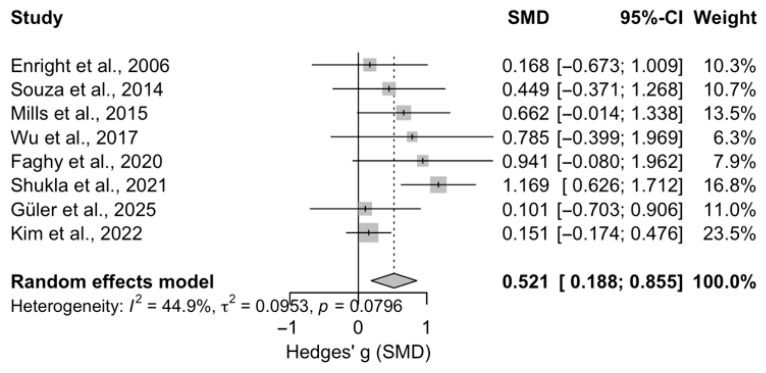
Forest Plot of the Effect of Inspiratory Muscle Training on Diaphragm Thickness [23,24,25,26,27,28,29,30].

According to the random-effects model, IMT significantly increased diaphragm muscle thickness in healthy adults (Figure 4).

Based on Figure 4, the random-effects meta-analysis including eight studies demonstrated a significant overall effect (Hedges’ g = 0.521, SE = 0.170, 95% CI [0.188, 0.855], z = 3.07, *p* = 0.002). The estimated heterogeneity was moderate (τ^2^ = 0.0953, I^2^ = 44.9%), and the test of heterogeneity was not statistically significant (Q(7) = 12.71, *p* = 0.080), suggesting that the variability among studies was moderate and that the pooled effect size was relatively consistent across the included studies.

### 3.8. Subgroup Analyses

**Figure 5:**
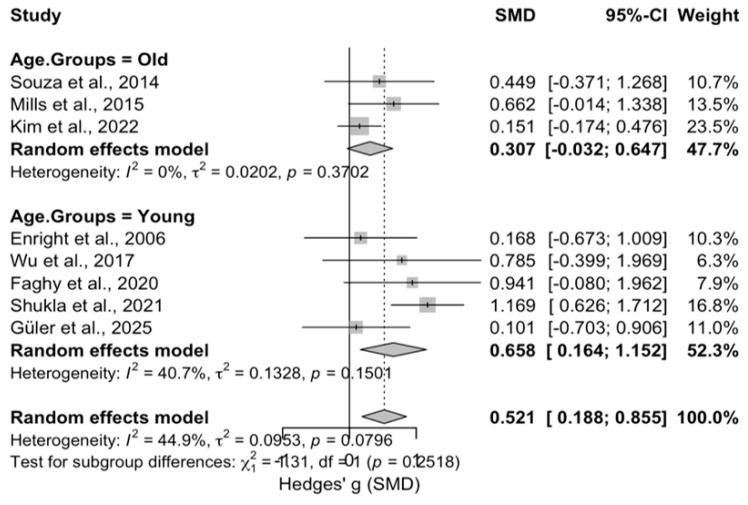
Forest Plot of Subgroup Analysis by Age Groups [23,24,25,26,27,28,29,30].

**Figure 6:**
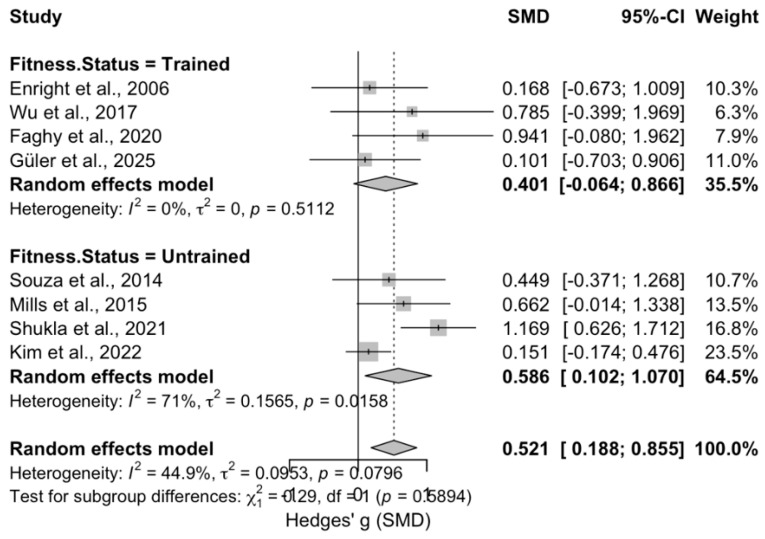
Forest plot of Subgroup Analysis by Training Status [23,24,25,26,27,28,29,30].

**Figure 7:**
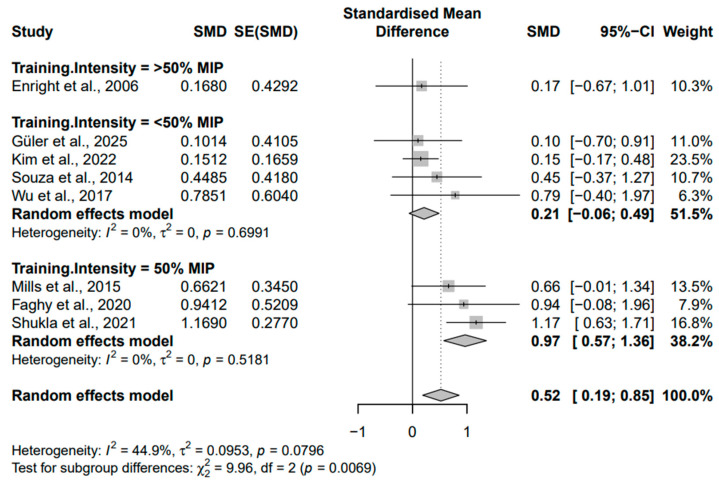
Forest plot of Subgroup Analysis by Training Intensity [23,24,25,26,27,28,29,30].

Subgroup analyses were conducted on diaphragm muscle thickness (mm), stratified by fitness status (untrained vs. trained) and inspiratory muscle training intensity (<50% of MIP, 50% of MIP, or >50% of MIP, according to the individual studies included in the meta-analysis), and were used to create forest plots. In addition, subgroup analyses were conducted on diaphragm muscle thickness based on age (old (>45 years) and young (<45 years)) (Figure 5, Figure 6 and Figure 7).

The subgroup analysis based on age group revealed no significant moderating effect (Hedges’ g = −0.318, 95% CI [−0.953, 0.317], *p* = 0.327; Q-between = 0.96, *p* = 0.327). The young subgroup demonstrated a significant effect (Hedges’ g = 0.658, SE = 0.226, 95% CI [0.164, 1.152], z = 2.97, *p* = 0.003), while the old subgroup did not reach statistical significance (Hedges’ g = 0.307, SE = 0.232, 95% CI [−0.032, 0.647], z = 1.53, *p* = 0.126). These results indicate that both groups benefited from the intervention; however, the effect was statistically significant only among younger participants.

According to Figure 6, the subgroup analysis based on fitness status revealed no significant moderating effect on the overall intervention outcome (Hedges’ g = 0.185, 95% CI [−0.489, 0.859], z = 0.54, *p* = 0.589; Q-between = 0.29, *p* = 0.589). The trained subgroup demonstrated a non-significant small-to-moderate effect size (Hedges’ g = 0.401, SE = 0.233, 95% CI [−0.064, 0.866], z = 1.72, *p* = 0.086). In contrast, the untrained subgroup showed a significant moderate improvement (Hedges’ g = 0.586, SE = 0.233, 95% CI [0.102, 1.070], z = 2.52, *p* = 0.012). However, the between-group difference was not statistically significant, suggesting that training status did not meaningfully moderate the intervention’s effects. Both trained and untrained participants benefited, though the effect was significant only in the untrained group.

In the subgroup analysis based on training intensity, the largest and statistically significant effect was observed in studies using 50% MIP (k = 3; Hedges’ g = 0.97, 95% CI [0.57–1.36]). In contrast, studies employing <50% MIP (k = 4) showed a small and non-significant effect (Hedges’ g = 0.21, 95% CI [−0.06–0.49]), while the single study using >50% MIP reported a small and non-significant effect as well (Hedges’ g = 0.17, 95% CI [−0.67–1.01]). The test for subgroup differences was significant (Q = 9.96, *p* = 0.0069), indicating that training intensity moderated the effect size. These results suggest that 50% MIP produces the strongest and statistically significant effect, whereas <50% and >50% MIP show small, non-significant effects.

### 3.9. Meta-Regressions

In this study, to better understand the origins of heterogeneity, meta-regressions were performed on baseline lung volumes and inspiratory muscle strength (Forced Vital Capacity (FVC), Forced Expiratory Volume in one second (FEV_1_), Maximal Inspiratory Pressure (MIP), and Maximal Expiratory Pressure (MEP)), age, and IMT intensity. These variables were meta-regressed in a random-effects meta-regression model in R (v. 4.4.2). Table 4 presents the results of the overall model meta-regressions.

**Figure 8:**
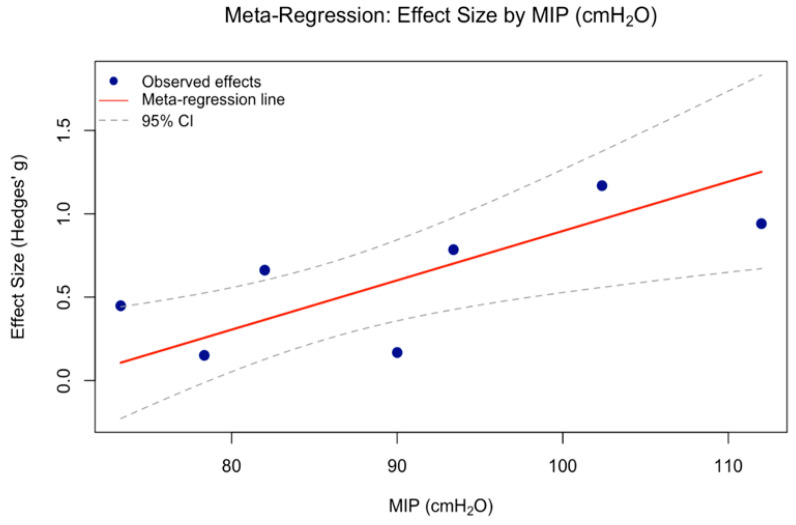
Random Effect Univariate Meta Regression between Baseline MIP Level and Effect Size.

According to Table 4, among the examined moderators, only baseline inspiratory muscle strength (Maximal Inspiratory Pressure value (MIP, cmH_2_O)) significantly predicted the intervention effect (β = 0.030, 95% CI [0.009, 0.050], *p* = 0.004), indicating that individuals with higher baseline MIP demonstrated greater improvements (Figure 8). Other variables -including baseline FEV_1_ (L), FVC (L), MEP (cmH_2_O), mean age (years), duration (weeks), intensity (% of MIP), and training frequency (per week)-did not show significant moderating effects (*p* > 0.10).

## 4. Discussion

This systematic review and meta-analysis investigated the effects of IMT on diaphragm muscle thickness. Previous studies have explored the impact of IMT on diaphragm thickness only in stroke patients [9,15,17]; no meta-analyses have targeted healthy individuals and/or athletes. In this respect, the present study represents the first systematic attempt to evaluate the effects of IMT on diaphragm muscle thickness in these specific groups. While the present systematic review and meta-analysis provide novel insights, it is not without limitations. A key constraint is the scarcity of research involving healthy older adults; only three of the included trials enrolled participants aged 45 or older. Furthermore, the literature demonstrates a significant gender imbalance, as the predominant focus on male populations precluded any meaningful analysis of sex/gender-based differences. In addition, moderate heterogeneity makes it difficult to generalize the results to a healthy population. Despite these limitations, the study’s results are significant because it is the first comprehensive synthesis focusing on the effect of IMT on diaphragm thickness in healthy adults and athletes.

The main finding of the study is that IMT leads to a significant hypertrophic adaptation in the diaphragm (Hedges’ g = 0.521; *p* < 0.05) (Figure 4). Subgroup analyses indicate that it was associated with a statistically significant effect on diaphragmatic hypertrophy. No significant differences were observed between age groups. Both younger and older participants benefited from IMT, but the effect was statistically significant only in the younger group. Similarly, there was no significant difference in hypertrophic responses between trained and untrained individuals. Regression analysis identified baseline MIP as the only significant predictor of diaphragmatic hypertrophy, while FEV_1_, FVC, MEP, age, and training parameters (duration, frequency, and intensity) were not statistically significant. Although effect sizes suggested potential advantages in younger, untrained individuals, the absence of significant subgroup differences precludes a conclusion of superiority. Numerical trends indicate that more complex models may help capture these variations. IMT performed at 50% of MIP supports diaphragmatic hypertrophy regardless of age or training status.

Subgroup analyses stratified by age further demonstrated that IMT increased diaphragm thickness in younger individuals (Hedges’ g = 0.658, *p* = 0.003). In contrast, the effects were attenuated or statistically nonsignificant in older adults. The absence of a significant between-group difference (*p* = 0.126) suggests that the hypertrophic response of the diaphragm might be independent of age (Figure 5). This pattern may reflect age-related changes in mechanisms of muscle adaptation. As age increases, activation of the mammalian target of rapamycin complex 1 (mTORC1) pathway tends to decline, while 5′ adenosine monophosphate-activated protein kinase (AMPK) activity rises [32,33,34]. Moreover, reductions in motor unit number, neuromuscular transmission efficiency, and anabolic sensitivity can limit the hypertrophic potential of respiratory muscles, such as the diaphragm. This effect may be more pronounced compared to other skeletal muscles [35]. While these mechanisms may help explain the observed pattern, current findings do not support a definitive difference between age groups.

Another subgroup analysis revealed that although the untrained subgroup showed a significant moderate improvement (Hedges’ g = 0.586, *p* = 0.012), the between-group difference was not statistically significant (*p* = 0.589), suggesting that training status did not meaningfully moderate the intervention’s effects (Figure 6). Hypertrophic response of the diaphragm appeared to be independent of an individual’s training status.

This aligns with evidence that the diaphragm may respond more effectively to specific inspiratory loading than to general exercise stimuli. Illi et al. [36] reported that respiratory muscle training produces distinct adaptations, often absent in general physical activity. From a mechanistic perspective, prior skeletal muscle training can induce anabolic resistance via pathways including AMPK activation and mTORC1 suppression [37,38]. However, such anabolic resistance may not be uniform across the respiratory musculature, which appears to exhibit distinct regulatory behavior. Witt et al. (2007) showed that IMT attenuates the respiratory metaboreflex [39], thereby improving functional efficiency, whereas Sheel (2002) emphasized the distinct adaptive profile of respiratory muscles compared with limb skeletal muscles [40]. Supporting these mechanistic insights, hypertrophic responses to IMT have been documented in both untrained populations (e.g., Souza et al., Enright et al. [23,24]) and in resistance-trained athletes, as reported by Çelikel et al. (2025) and Güler et al. (2025), where control groups with matched training histories failed to show similar adaptations [30,41]. These findings support the view that IMT can elicit diaphragm hypertrophy regardless of an individual’s training status.

Regarding training intensity, the hypertrophic response was significantly greater with IMT at 50% MIP than at either lower or higher intensities. Notably, IMT with 50% of MIP (Hedges’ g = 0.97) yielded more consistent outcomes compared to those of <50% and >50% of MIP (Hedges’ g = 0.21, *p* = 0.69; Hedges’ g = 0.17, respectively) (Figure 7).

This finding may reflect the diaphragm’s distinct physiological profile as a fatigue-resistant and continuously active muscle. It appears to respond more favorably to training loads at approximately 50% of MIP, consistent with its endurance-based function. Unlike skeletal muscles, where higher intensities are typically associated with greater hypertrophy [42,43], the diaphragm appears to respond favorably to lower-intensity training [36,44]. Although 50% MIP emerged as the most effective intensity level in our meta-analysis, this value has often appeared in the literature under broader classifications such as “moderate,” “submaximal,” or even “lower” intensity [30,36,44,45,46]. Notably, many of these studies report significant physiological adaptations within ranges that encompass 50% of MIP (e.g., 40–60%, 45–55%), underscoring the relevance of this intensity level. Given the lack of consensus on a standardized intensity classification system for IMT, our findings help anchor these ranges quantitatively around a specific and effective loading point.

Among the variables examined in the meta-regression, a significant association was observed between baseline MIP (cmH2O) and diaphragm thickness (see Table 4). In other words, individuals with a higher initial MIP value are more likely to exhibit greater diaphragm hypertrophy following IMT. Age, training status, and other pulmonary function markers (e.g., FEV_1_, FVC, MEP) were also included in the model; however, none showed a significant association with diaphragm hypertrophy [4,13,45].

Lastly, our meta-regression examining IMT intensity and frequency as continuous predictors did not identify a significant association with diaphragm hypertrophy (*p* = 0.966, *p* = 0.582). However, subgroup analyses indicated that protocols using 50% of the MIP elicited more consistent hypertrophic responses. The findings suggest that diaphragm adaptations may not follow a strictly linear dose–response relationship. This pattern aligns with the diaphragm’s physiological profile as a highly fatigue-resistant muscle [4]. While intensity and frequency alone did not emerge as significant predictors in our regression, their interaction with individual characteristics (such as baseline MIP) may play a more complex role. This underscores the potential value of adopting multifactorial modeling in future studies. Consistent with this view, IMT has been shown to enhance exercise tolerance through respiratory muscle adaptations [4], and greater inspiratory strength has been associated with thicker diaphragmatic profiles and improved pulmonary function in healthy individuals [2,47]. Taken together, these findings suggest that initial inspiratory capacity may serve not only as a physiological baseline but also as a key modulator of adaptive hypertrophic responses to IMT.

Practical Applications

While hypertrophic responses varied across populations, the largest effect sizes were observed in younger, untrained adults—suggesting that these groups may benefit more from IMT in terms of diaphragm adaptation. Although statistical significance was not reached in some subgroup analyses, the consistently large effect sizes suggest a practical advantage for this demographic. For coaches and trainers, especially in sports that require sustained respiratory effort (e.g., endurance events, combat sports), applying IMT at approximately 50% MIP may be a safe and effective threshold for inducing structural adaptations in the diaphragm. Our findings indicate that this loading level aligns well with the diaphragm’s endurance-oriented physiology and produces consistent hypertrophic outcomes without excessive fatigue. This also has implications for respiratory readiness in professions where physical performance under pressure is critical, as diaphragm strength may directly affect task performance and fatigue resistance.

Clinical Implications and Future Perspectives

Beyond athletic settings, the clinical relevance of diaphragm hypertrophy warrants attention. Diaphragm adaptations are valuable not only for athletes but also for frontline professionals and patients at risk of respiratory weakness. In healthy adults, diaphragm thickness is approximately 0.18 cm at FRC and 0.48–0.56 cm at TLC, with a thickening fraction of 170–204% [48]. Carrillo-Esper et al. (2016) [49] reported average values of ~0.19 cm in men and ~0.14 cm in women, while Boon et al. (2013) [50] considered <0.15 cm as abnormal. Brown et al. (2013) showed that elite powerlifters exhibited significantly greater diaphragm thickness than control subjects [47]. Although the functional consequences of increased diaphragm thickness remain uncertain, even modest morphological improvements could support better respiratory efficiency in both clinical and high-demand settings. For physiotherapists and clinicians, this suggests that tailored inspiratory muscle training protocols may be a valuable tool to enhance respiratory robustness in otherwise healthy individuals. However, the functional significance of these structural adaptations remains incompletely elucidated, particularly with respect to performance, endurance, and respiratory morbidity.

Future studies should aim to establish normative diaphragm thickness thresholds associated with meaningful functional outcomes across diverse populations. Closely related to this is the need to determine the minimal clinically significant difference (MCID) in diaphragm thickness that translates into measurable health or performance benefits. Identifying such thresholds would greatly help define clinically relevant training outcomes for both medical and athletic populations. These situations are critical given the growing use of IMT in both clinical and athletic contexts, where structural adaptations are often assumed to reflect functional gains—an assumption that remains unverified. Moreover, although our analysis identified 50% of maximal inspiratory pressure (MIP) as the most effective training intensity, the literature is inconsistent in labeling this load as “moderate,” “submaximal,” or “low.” This lack of standardized intensity classification in IMT protocols complicates cross-study comparisons and hinders the development of evidence-based guidelines. Future research should therefore focus on defining and standardizing %MIP-based intensity zones for IMT.

Additionally, sex- and age-specific adaptations to IMT remain underexplored—especially among older adults and female participants, who are underrepresented in the current literature. Ultimately, the broader question of whether increased diaphragm thickness is necessary—or even advantageous—for athletes, frontline professionals, or clinical populations remains open. While our findings offer a foundation, future longitudinal and mechanistic studies are required to determine functional thresholds and long-term implications.

Limitations

Despite the novel insights this meta-analysis offers, several limitations should be acknowledged. First, the number of studies involving healthy older adults was notably limited, with only three trials including participants aged 45 or older. Additionally, most included studies focused on male participants, precluding analysis of sex-specific effects. Moderate heterogeneity observed across studies suggests that differences in protocols, participant profiles, and measurement methods may affect the consistency of outcomes. The presence of heterogeneity, along with the scarcity of studies with larger and more diverse sample sizes, limits the generalizability of the results to other populations. In addition, the limited number of studies focusing on elite athlete populations may limit the precision of results for this subgroup. These limitations highlight the need for future high-quality randomized controlled trials with more heterogeneous samples to better understand the impact of IMT on diaphragm hypertrophy.

## 5. Conclusions

This systematic review and meta-analysis demonstrate that an IMT program with 50% MIP can significantly increase diaphragm muscle thickness, independent of fitness status or age, in healthy adults. Moreover, our findings provide compelling evidence that baseline inspiratory muscle strength, specifically MIP, may serve as a stronger predictor of diaphragmatic hypertrophy than training intensity alone. These results offer actionable guidance for optimizing IMT protocols in both health and performance contexts. For athletes, IMT sessions may improve ventilatory efficiency, delay fatigue, and reduce respiratory limitations during high-intensity exercise. For healthy non-athletes, IMT may be applied as a preventive strategy to preserve diaphragm function and mitigate age-related decline. Although this analysis focused on healthy populations, the adaptations observed suggest potential benefits for clinical groups as well. IMT may therefore be explored as a non-invasive approach to support diaphragm function in these populations. Future studies should further refine IMT protocols and test their effectiveness across diverse populations.

## Figures and Tables

**Table 1:** Quality of included trials based on the Physiotherapy Evidence Database (PEDro) criteria and scores.

Study	EC	I	II	III	IV	V	VI	VII	VIII	IX	X	Total
Enright et al., 2006 [23]	√	√	√	√	√	×	×	√	×	√	√	8
Souza et al., 2014 [24]	√	√	√	√	√	×	√	√	×	√	√	9
Mills et al., 2015 [25]	√	√	×	×	√	×	×	√	×	√	√	6
Wu et al., 2017 [26]	√	√	×	×	√	×	×	√	×	√	√	6
Faghy et al., 2021 [27]	√	√	√	√	×	×	×	√	×	√	√	7
Shukla et al., 2021 [28]	√	√	√	√	√	×	×	√	×	√	√	8
Kim et al., 2022 [29]	√	√	×	√	√	√	×	√	×	√	√	8
Güler et al., 2025 [30]	√	√	√	×	√	×	×	√	×	√	√	7
Total												7.37

Abbreviations: EC: eligibility criteria; I: random allocation; II: concealed allocation; III: baseline comparability; IV: blinding of subjects; V: blinding of researchers/evaluators; VI: blinding of assessors; VII: measure of at least one key outcome obtained from more than 85% of subjects initially allocated to groups; VIII: intention to treat; IX: comparison results between groups; X: measured at least one key outcome at two time points; √: criterion is present; ×: criterion is missing.

**Table 2:** Characteristic of Inspiratory Muscle Training and Outcomes.

Study	Population	Study Design	Training Protocol	Duration	Ultrasonography Procedure	Main Outcomes
Enright et al., 2006 [23]	20 moderately trained adults, Experimental: 10, Control: 10; F: 11 M: 9; Age: 18–25 years	Single-center controlled study	IMT: 80% of MIP × 6 sets; the rest time between repeats was gradually lowered from 60 s to 45, 30, 15, 10, and 5 s throughout each set. CON: No intervention.	8 weeks	7.5 MHz linear probe, 8th–9th intercostal, anterior-middle axillary line, left lateral decubitus position	12% increased diaphragm thickness in the experimental group ↑ (*p* < 0.05). No increase in diaphragm thickness was observed in the control group.
Souza et al., 2014 [24]	22 elderly women, Experimental: 12, Control: 10; Age: 60–80 years	RCT	IMT: 40% of MIP × 8 sets, CON: Same protocol as IMT, but without a resistive load.	8 weeks	7.5 MHz linear probe, 8th–9th intercostal, anterior-middle axillary line, left lateral decubitus position	After IMT, diaphragm thickness increased by 11% in the experimental group (*p* = 0.001).
Mills et al., 2015 [25]	34 healthy older adults, Experimental: 17, Control: 17; F: 8 M: 9; Age: 65–75 years	RCT	IMT: 50% of MIP, 2 × 30 breaths, and incremental intensity. CON: Same protocol as IMT, but without a resistive load.	8 weeks	NR	After IMT, diaphragm thickness at residual volume 38% increased ↑ (*p* = 0.03)
Wu et al., 2017 [26]	10 healthy male tennis players, Experimental: 5, Control: 5; Age: 18–25 years	RCT	IMT: 30% of MIP with 2 × 30 breaths. CON: Same protocol as IMT, but without a resistive load.	6 weeks	7.5 MHz linear probe, 8th intercostal midaxillary line.	Diaphragm thickness was significantly (*p* < 0.05) improved in the IMT group (pre (2.3 ± 0.3 mm) vs. post (2.8 ± 0.1 mm)).
Faghy et al., 2021 [27]	23 recreationally trained runners, Experimental-1: 1: 8, Experimental-2: 8, Control: 7; F: 10 M: 13; Age: 27–45 years	RCT	Experimental-1: IMT 50–60% of MIP with 2 × 30 breaths, Experimental-2: HIIT training with flow-resistive mask CON: Same protocol as IMT, but without a resistive load.	6 weeks	L17–5 MHz linear probe, 7th–10th intercostal space, right mid-axillary line	Diaphragm thickness was significantly (*p* = 0.032) improved in the IMT group by 9.5 ± 3.4% from pre (1.8 ± 0.2 mm) vs. post (2.0 ± 0.2 mm) [absolute change = 0.2 ± 0.2 mm, effect size d = 0.73].
Shukla et al., 2021 [28]	60 healthy young adults, Experimental: 30, Control: 30; F: 41, M: 19; Age: 22–23 years	RCT	IMT: 50% of MIP with 2 × 30 breaths. CON: Same protocol as IMT, but without a resistive load.	8 weeks	7.5 MHz linear probe, 8th–9th intercostal, right middle axillary line, left lateral decubitus position.	Diaphragm thickness was significantly (*p* < 0.05) increased in the IMT group (pre (1.83 ± 0.27) vs. post (2.15 ± 0.28)). In contrast, the control group showed no difference.
Kim et al., 2022 [29]	80 active community-dwelling older men, Experimental-1-2-3-4: 20 Age: 73–76 years	Pre-post design	Experimental-1: 40% of MIP with 10 sets × 10 breaths in rehabilitation center via positive expiratory pressure device, Experimental-2: 40% of MIP with 10 sets × 10 breaths in rehabilitation center, Experimental-3: 40% of MIP with 10 sets × 10 breaths at home via positive expiratory pressure device, Experimental-4: 40% of MIP with 10 sets × 10 breaths at home.	8 weeks	12 MHz linear probe, 8th–9th ribs of the anterior and middle axillary line, supine position.	In the Experimental-2 group, no significant difference was observed in diaphragm muscle thickness in the post-test compared to the pre-test results (change from baseline 0.2 ± 1.27, *p* = 0.527).
Güler et al., 2025 [30]	22 male bodybuilders Experiment: 11, Control: 11, Age: 22.45–24.82 years	RCT	IMT: 40% of MIP with 30 breaths. CON: No IMT intervention.	4 weeks	12 MHz linear probe, intercostal space between 10th and 11th ribs, middle axillary line, supine position.	Diaphragm thickness 19.46% more improvement in the IMT group (28.69%, *p* < 0.001) compared to the control group (9.21%, *p* = 0.019).

Abbreviations: F: Female; M: Male; IMT: Inspiratory Muscle Training; NR: Not Reporting, MHz: Megahertz, CON: Control group; RCT: Randomized controlled study; MIP: Maximal Inspiratory Pressure; HIIT: High Intensity Interval Training.

**Table 3:** Heterogeneity Test Results.

Cochran’s Q	Hedges’ g (95% CI)	dF	*p*	I^2^ (%)	tau^2^
12.71	0.52 (0.19–0.85)	7	0.080	45.2	0.095

**Table 4:** Meta Regression Outputs.

Variables	k	τ^2^	I^2^ (%)	Estimate (β)	SE	95% CI (Lower–Upper)	z	*p*	R^2^
Baseline FEV_1_ (L)	6	0.0580	34.95	0.532	0.333	−0.121–1.185	1.60	0.110	%0
Baseline FVC (L)	6	0.1237	56.22	0.240	0.329	−0.405–0.885	0.73	0.466	%0
Baseline MIP (cmH_2_O)	7	0.0000	0.00	0.030	0.010	0.009–0.050	2.87	0.004 **	%100
Baseline MEP (cmH_2_O)	6	0.1183	50.51	0.013	0.018	−0.022–0.048	0.74	0.460	%0
Mean Age (years)	8	0.0765	35.60	−0.007	0.007	−0.020–0.007	−0.97	0.333	%0
Duration (weeks)	8	0.1146	50.17	0.047	0.132	−0.211–0.305	0.36	0.719	%0
Intensity (% of MIP)	8	0.1169	50.06	−0.001	0.014	−0.028–0.027	−0.04	0.966	%0
Frequency (per week)	7	0.0854	34.35	0.076	0.139	−0.195–0.348	0.55	0.582	%0

Note: ** *p* < 0.01.

## Data Availability

Data are available for research purposes upon reasonable request to the corresponding author.

## Supplementary Materials

The following supporting information can be downloaded at: https://www.mdpi.com/article/10.3390/medicina62030609/s1. PRISMA 2020 Checklist.
